# The Influence of the Variable Wettability Characteristics of Layers on the Transport of Nanoparticles in the Context of Drug Delivery in Skin Structures

**DOI:** 10.3390/ijms25094665

**Published:** 2024-04-25

**Authors:** Mariola M. Błaszczyk, Łukasz Przybysz, Aleksandra Budzyń

**Affiliations:** Faculty of Process and Environmental Engineering, Department of Chemical Engineering, Lodz University of Technology, 213 Wolczanska St., 90-924 Lodz, Poland

**Keywords:** nanoparticles, drug delivery, diffusion, transdermal transport, skin layers

## Abstract

The rapid development of nanotechnology has offered the possibility of creating nanosystems that can be used as drug carriers. The use of such carriers offers real opportunities for the development of non-invasive drug delivery through skin structures. However, in addition to the ability to create suitable nanocarriers, it is also necessary to know how they move through dermal layers. The human skin consists of layers with different wettability characteristics, which greatly complicates how introduced substances move through it. In this work, an experimental study of the diffusion process of nanoparticles through partitions with different wettability properties was carried out. Conventional diffusion tests using Franz chambers were used for this purpose. We quantified how the wettability of the barrier, the number of layers, and their mutual configuration affect the transport of nanoparticles. Based on the results, an analysis of the phenomena taking place, depending on the wettability of the partition, was carried out. A model relationship was also proposed to determine the effective diffusion coefficient, taking into account the influence of the wettability and porosity of the barrier.

## 1. Introduction

The transport of dispersion systems, especially in small, micro-, or nanostructures, plays a fundamental role in many fields of science and industry such as medicine, pharmaceuticals, and oil extraction [1]. One of the greatest challenges at present is the non-invasive introduction of active substances into the human body. This will make it possible to cease the use of outdated injection methods, which are not only painful but can also cause cross-infection, which directly threatens human life. The development of nanotechnology and the ability to create increasingly complex drug carriers at the nanoscale offers increasingly realistic prospects for meeting this challenge. However, in the absence of sufficient knowledge of the transport mechanisms of such systems through the human skin structure, a deeper consideration of these issues is necessary.

**Figure 1:**
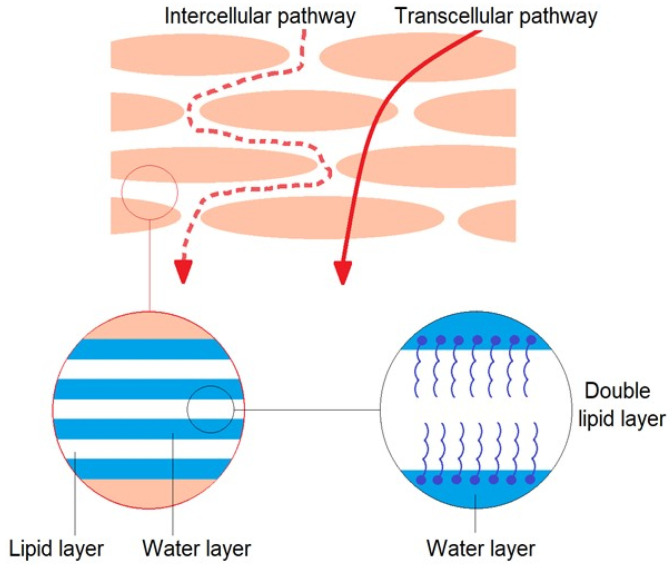
Intercellular and transcellular pathway.

The human skin consists of layers, which are characterized not only by different structures but also by varied lipophilicity properties [2]. Due to the tight structure of keratinocytes and the laminar arrangement of lipids surrounding the cells, the most difficult layer to overcome is the outer layer of the skin—the stratum corneum [3]. This layer in its structure consists of alternating lipid layers and aqueous layers [4] (See Figure 1).

The changing wettability of the medium greatly complicates the ability to transport the introduced compounds. For this reason, active substances are often placed on (or in) so-called carriers and, in this form, are transported to select layers of the skin [5]. The simplest carriers of active substances are nanoparticles, which provide a matrix for many types of compounds [6]. Rapid advances in nanotechnology in recent years have contributed to the possibility of creating nanoparticles with well-defined parameters adapted for medical purposes [7,8]. However, in order to use the full potential of nanoparticles as non-invasive drug carriers, it is not enough to produce them; it is also necessary to understand the mechanisms of their transport through the structure of the skin [9].

The most difficult skin layer to bypass is the stratum corneum; for this reason, in the literature, the study and modeling of various systems’ transport through this layer is considered [10,11,12]. Using the brick wall model as a method to represent the structure of the skin has been adopted. In this model, skin cells represent the bricks of a wall, while the cement of the wall is the lipid matrix [13,14]. There are many theoretical and experimental works in the literature describing the transport of various substances through this cement, i.e., through the intercellular pathway. Various modifications of Fick’s first or second law have been used to describe such flows, and the most important parameter that has been used to determine these is the diffusion coefficient *D* [15,16].

**Figure 2:**
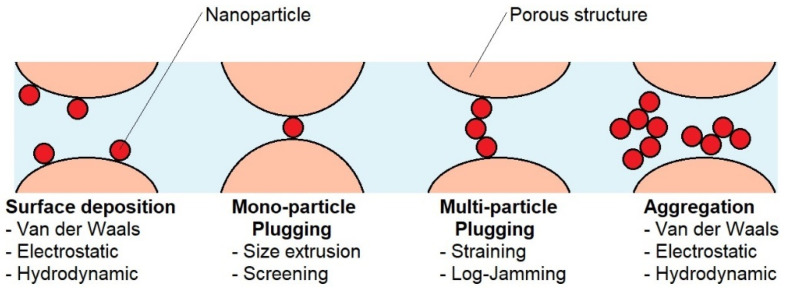
Mechanisms of retaining particles while flowing through a porous structure.

However, the relationship above is a major simplification and does not capture the complex phenomena occurring during the transport of drug carriers, e.g., nanoparticles [17,18]. The transport of nanoparticles through the stratum corneum structure depends on their interactions with skin cells. In such a system, the existence of three “phases” can be assumed: the solid phase of skin cells (corneocytes), the dispersed phase (nanoparticles), and the phase in which nanoparticles are dispersed. The properties of each phase affect how the nanoparticles move. Assuming that the driving force of the transport process is the concentration gradient, nanoparticles will diffuse. However, the diffusion process will be perturbed and significantly different than if it occurred in a single-phase liquid. Nanoparticles moving through the stratum corneum structure can undergo adsorption processes and can also form aggregates. They can plug into small spaces as single particles and into larger ones as aggregates, causing multi-particle plugging [19]. All these mechanisms and the reasons for their formation are shown schematically in Figure 2.

Which phenomenon, when it occurs, and how it affects the overall transport of nanoparticles are fundamental issues when describing the transport of nanosystems through the stratum corneum. The modeling of this process is also a challenge, due to its difficult-to-predict nature. The wettability of the individual phases is of great importance, as it determines all surface phenomena [20,21,22]. The adsorption process occurs differently with hydrophilic nanoparticles on hydrophilic membranes in comparison to when the membranes exhibit hydrophobic properties.

**Figure 3:**
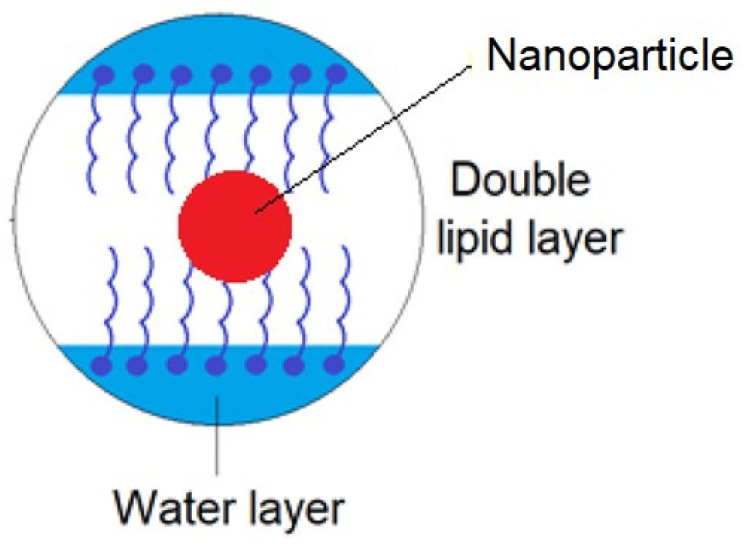
Retention of a nanoparticle in a double lipid layer.

In addition, given the existence of a double lipid layer in the skin structure, nanoparticles moving in the channels between skin cells may encounter hydrophobic and hydrophilic zones. Transport through such zones can be difficult, and nanoparticles can become trapped between these layers, as presented in Figure 3. Nanoparticles entrapped in such zones do not participate in the flow, which affects the global permeability of the substance [23].

Considering the complex problem of variable wettability and its influence on the phenomena occurring during nanoparticle diffusion, flow modeling is difficult. The literature lacks both experimental data and correlations derived from them to show how variable wettability characteristics of layers can affect the nanoparticle diffusion process. This would provide valuable input for the global modeling of nanocarrier transport in dermal structures and contribute to the development of effective, non-invasive drug application methods. Guided by these motivations, experimental studies were conducted to capture the effect of the dependence of porous layer wettability variation on the nanoparticle diffusion process. This allowed us to determine how the occurrence of zones with different wettability characteristics can be taken into account during the description and modeling of transport. Thanks to the work carried out, it becomes possible to determine the diffusion coefficient for nanoparticles moving through layers of varying wettability. The possibility of modifying existing models describing drug delivery was proposed so that the description of the diffusive transport of nanoparticles will take into account the effect of varying hydrophilicity on the speed and intensity of the process.

## 2. Results

### 2.1. Transport of NanoCu through Filters with the Same Wettability

For comparison purposes, diffusion tests of copper nanoparticles through the same wettability barriers were carried out in the first stage of this study. The amount of particles that were diffused from the donor chamber to the acceptor chamber through the filter was presented as a dimensionless quantity, representing the ratio of concentrations, according to the following relation:

(1) $$N=\frac{c_{a}}{C_{dp}}$$

where *N*—dimensionless amount of nanoparticles in solution [-], *c_a_*—concentration of acceptor fluid after a given time [ppm], and *c_dp_*—concentration of the donor fluid [ppm] at the time *t* = 0 s.

**Figure 4:**
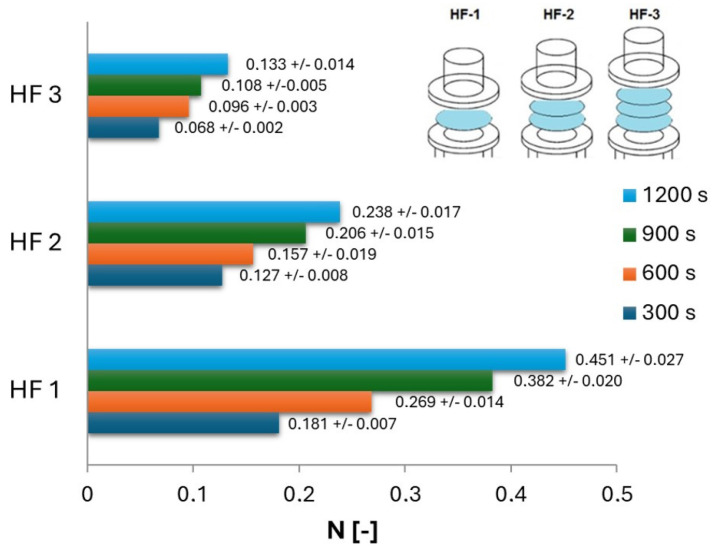
Amount of nanoCu in the acceptor fluid relative to the diffusion time for different layers of hydrophilic filters (HF).

**Figure 5:**
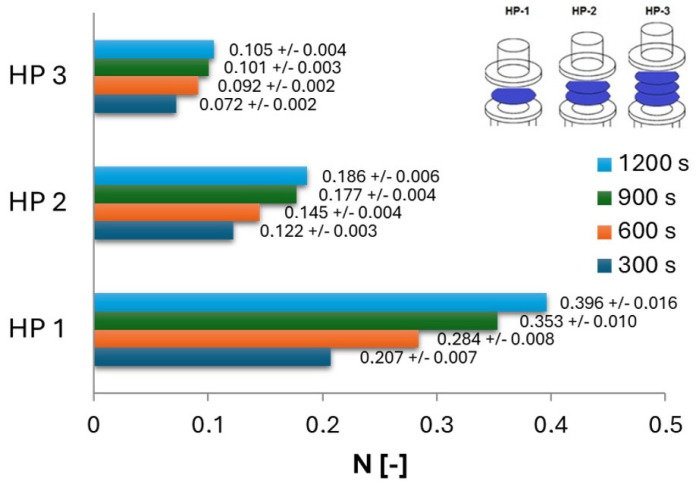
Amount of nanoCu in the acceptor fluid relative to the diffusion time for different layers of hydrophobic filters (HP).

Figure 4 presents the measured values of the amount of nanoCu nanoparticles in solution after the diffusion process at a given time for individual layers of hydrophilic partitions (HF), while Figure 5 presents the corresponding *N* values for individual layers of hydrophobic filters (HP). As expected, as the diffusion time increased, the *N* of particles (that underwent diffusion) increased; however, diffusion was the most intense in the first stage of the process, when the concentration difference was the greatest. After a time of 300 s, more than 50% of nanoCu that underwent diffusion was recorded compared to the value obtained after 1200 s.

As the process time increased, the increase in *N* values lessened in each case studied. The highest *N* values were, of course, recorded for single filters, with the amount of particles in the acceptor solution decreasing with the application of another layer. However, it is noteworthy that the membranes used had mesh diameters of 1 micrometer, while the particles moving through were of the nanometer order. Despite such a large difference in size, the filters used provided a high barrier to nanoparticle transport even with a single filter layer. The use of a mixing procedure greatly intensified the process, but the tendencies remained.

The use of double hydrophilic baffles versus single ones resulted in a reduction by half of the *N* value recorded after 1200 s of the process. The use of triple HF membranes, on the other hand, resulted in a decrease of as much as 70% of the dimensionless number of particles in solution relative to single membranes. For hydrophobic barriers, the trends are similar, except that the use of triple filter layers resulted in as much as a more than threefold decrease in *N* values.

The measured values of the dimensionless number of particles in solution for hydrophilic filters were, in each case, greater than the corresponding values for hydrophobic filters. Comparing the *N* values obtained for a total process time of 1200 s, when single HF barriers were used, the *N* values were 14% greater than those for HP barriers. When double layers were used, 28% higher values could be observed for HF filters, while 27% were observed with triple layers. This means that hydrophobic membranes are a greater obstacle to nanoCu diffusion than hydrophilic filters. This is due to the fact that the nanoCu used exhibited hydrophilic properties. When transporting hydrophilic particles through hydrophobic barriers, there are additional forces that are not present during transport through hydrophilic ones. These forces can affect interfacial phenomena among the filter–water–particle media.

### 2.2. Transport of NanoCu through Alternating Wettability Filters

**Figure 6:**
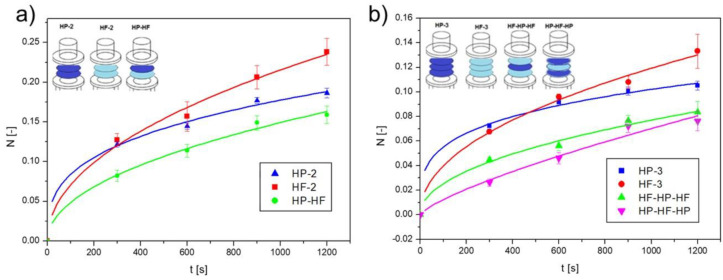
Amount of nanoCu in the acceptor fluid relative to diffusion time for (**a**) double partitions and (**b**) triple partitions.

The most relevant flow, from the perspective of the transport of drug carriers through complex biological structures, is flow through structures with variable wettability properties. In order to show the effect of changing wettability on the diffusion process of nanoparticles, layered filters of varying wettability were used as partitions. Tests with double filters were the starting point of the study. In Figure 6a, results for the diffusion process of nanoCu through double filters—one with hydrophobic properties and the other with hydrophilic properties (HP-HF)—are shown. For comparison, results for double hydrophobic barriers (HP-2) and double hydrophilic barriers (HF-2) are also presented. By contrast, Figure 6b shows the results for diffusion through triple partitions. The time dependence of N for hydrophobic (HP-3), hydrophilic (HF-3), and alternating hydrophilic–hydrophobic–hydrophilic (HF-HP-HF), and hydrophobic–hydrophilic–hydrophobic (HP-HF-HP) filters is presented.

From the graphs in Figure 6, it can be observed that for multiple hydrophobic filters, there was initially a larger amount of nanoparticles that underwent diffusion than in the case of hydrophilic partitions; however, with the duration of the process, a larger amount of nanoCu passed through the hydrophilic filters. However, the most surprising results are from the diffusion process of nanoparticles at partitions with mixed wettability characteristics. Here, much smaller amounts of nanoCu were observed in the acceptor chamber than for filters with the same wettability characteristics. For double baffles (HP-HF), the final (after t = 1200 s) *N* value was 15% lower than that achieved for filters (HP-2) and as much as more than 30% lower than for filters (HF-2). In the case of triple partitions, the results diverged even more from each other. In this case, however, the way the filters were arranged was also important. With the (HP-HF-HP) arrangement, the amount of nanoCu was more than 42% less than for hydrophilic filters. With the (HF-HP-HF) arrangement, the *N* value was 37% lower than for (HF-3). Thus, it turned out that the use of an alternating arrangement of filters with different wettability characteristics significantly reduced the diffusion process of nanoparticles. This had important implications for transport through biological structures, such as the skin, where zones of variable wettability can be observed. However, in order for the presented results to be analyzed in the context of transport through the skin, more detailed analyses of this phenomenon were needed.

**Figure 7:**
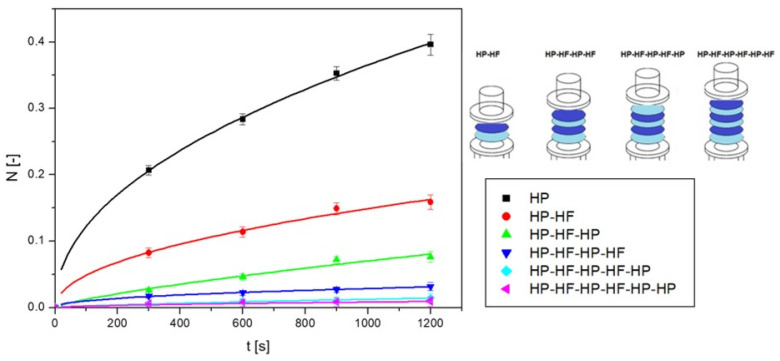
The amount of nanoCu in the acceptor fluid relative to the diffusion time for sequential alternating partitions.

Figure 7 shows the results of the change in the dimensionless amount of nanoparticles in solution versus time for sequential alternating layers. Each new layer caused the *N* value to decrease by almost half. In the case of using six layers, the results obtained were so small that it can be considered that the diffusion process under these conditions was inhibited.

**Figure 8:**
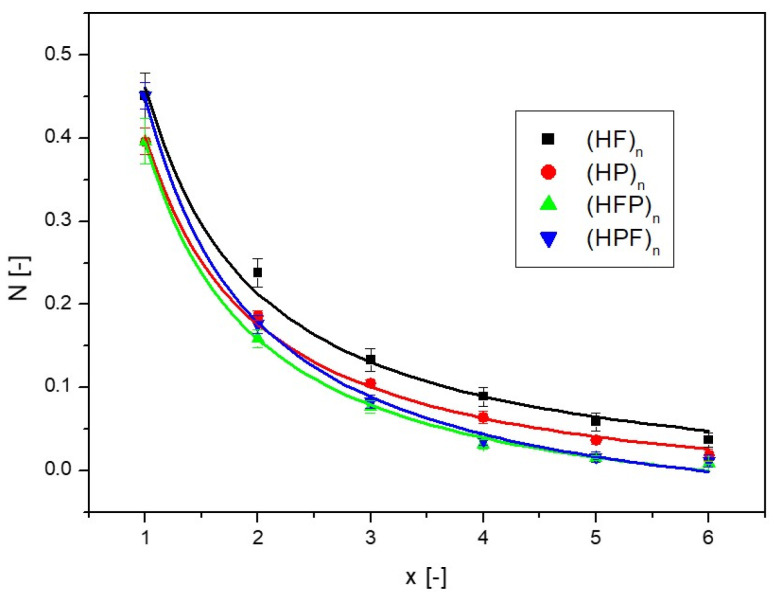
Amount of nanoCu in the acceptor fluid relative to the number of layers for the diffusion process for different types of partitions.

In order to compare the results achieved, the *N* values obtained for the final time (t = 1200 s) are presented for successive layers of partitions *x*, as shown in Figure 8. Since some difference in the results was observed due to the way the filters were arranged, the arrangement where the first filter was hydrophilic was named (HFP)_n_, while the arrangement where the first filter was hydrophobic was named (HPF)_n_. Subsequent layers of partitions with the same wettability characteristics were named (HF)_n_ for hydrophilic filters and (HP)_n_ for hydrophobic filters.

Obviously, the amount of nanoCu in the acceptor fluid decreased with the application of the next filter; however, if the filters were of alternating wettability, the decrease was much greater than for homogeneous hydrophilic and hydrophobic filters. The partition arrangement of (HPF)_n_ and (HPF)_n_ influences the amount of pre-founded nanoparticles in the case of a small number of filters; as more layers are added, the differences are blurred.

In order to describe the obtained results of the dependence of the amount of nanoCu over time, a correlation was proposed in the following form:

(2) $$N=a\;t^{n}$$

On the other hand, the description of the results of the dependence of *N* on the number of layers was made using the following relation:

(3) $$N=\frac{b}{x}-c$$

where *a*, *b*, *c*, and *n* are the parameters of the equation and, along with the coefficient of fitting R^2^, are provided in Table 1. Descriptions using the correlations above are shown as solid lines in Figure 6, Figure 7 and Figure 8.

## 3. Discussion

### 3.1. Description of the Transport Process of Nanoparticles through Partitions with Different Wettability Characteristics

In order to understand how transport through structures of different wettability occurs, it is necessary to take into account the changing conditions inside the structure as the nanoparticles move. Note the fact that wettability will directly affect the surface phenomena that can occur between the partition walls and the nanoparticles, such as adsorption. In the case of hydrophilic filters, hydrophilic nanoparticles will tend to adhere to the filter surface, while in the case of hydrophobic partitions, such particles will be repelled. This will cause, in the case of hydrophilic layers, nanoparticles to coat their surfaces. This will also result in some reduction in the number of particles passing through in the initial period (due to the occurrence of the trapping process), but after some time, when all the places where the particles could be trapped are filled, the transport will proceed undisturbed through almost the entire diameter of the channel, reduced only by the thickness of the layer of trapped nanoparticles. Given the mesh size of the partitions (micrometric) and the size of the nanoparticles, this reduction will be negligible.

In the case of hydrophobic layers, there will be no adsorption process on the surface, but the particles repelled by the surfaces will tend to flow through the center of the channel, resulting in some reduction in the active diameter of the channel through which the particles move. This reduction may be large enough to affect the total diffusion flux. This concept explains the initial relatively high number of particles passing through the membranes (no capture by the surface) observed during this study, compared to transport through hydrophilic filters. However, a globally lower amount of *N*, compared to HF filters, is caused by the action of particle repulsion from the walls.

The most interesting situation occurs when dealing with alternating layers. Hydrophilic layers will result in the attraction of particles and adsorption on their surface, while hydrophobic layers will repel particles from the surface. This repulsion of particles by HB layers will lead to the movement of particles in the area near HF layers; this accumulation of nanoparticles in these zones will result in enhanced adsorption, which, with sufficient particle accumulation, can be multilayered. The number of particles that manage to pass through the first HF layer will be reduced. In addition, they will move through the HB layer at a suitable distance from the surface. When they pass through the HB layer, they will be attracted again by the next hydrophilic layer, and there, they will undergo adsorption on the surface again. This will result in a global decrease in the number of particles that pass through the partition. Each successive alternating layer will intensify the described phenomena. The described particle behavior explains why the smallest number of particles passing through is observed at partitions with alternating wettability layers compared to homogeneous HB or HF partitions.

### 3.2. Mathematical Description of the Transport of Nanoparticles through Layers of Different Wettability—Determination of the Effective Diffusion Coefficient

Assuming that the driving force for the movement of the nanoparticles is the concentration difference, it can be assumed that the transport takes place by diffusion. The mass flux of diffusing nanoparticles *J* is thus equal to the following:

(4) $$J=D_{ef}\frac{c_{0}-c_{p}}{L}$$

where *D_ef_*—effective diffusion coefficient, *L*—path length (partition thickness) *c*_0_—initial concentration, and *c_p_*—concentration of nanoparticles that underwent diffusion.

The mass exchange flux can be defined as follows:

(5) $$J=\frac{m_{p}}{A\cdot t}$$

where *m_p_* is the mass of nanoparticles that were diffused through the septum from a given volume of liquid *V*, calculated on the basis of concentration, from the relation *m_p_ = c_p_·V*; knowing the diameter of the filter cross-section through which the diffusion process took place (0.02 m), it is possible to calculate its area *A* (0.000314 m^2^) and *t*—diffusion time.

The effective diffusion coefficient for diffusion through porous structures is a function of the diffusion coefficient *D* of the fluid in an unconfined fluid (no barrier) under given conditions and the porosity *ε* of the filter. However, as described above, during transport through structures of different wettability, a variety of surface phenomena can occur. Among these phenomena, one can expect adsorption, the capture of particles inside the filters, or the repulsion of nanoparticles by the surface of the partitions causing a reduction in the cross-section through which the particles flow. In such a situation, the actual porosity *ε_r_*, the actual space through which particles can diffuse, becomes smaller. It is therefore important to estimate how this actual porosity depends on the type and thickness of the partitions (number of layers).

It can be assumed that all surface phenomena depend on the total surface area the nanoparticles can contact—in other words, on the number of layers. The more layers, the more intense the phenomena. Assuming that when flowing through single layers, surface phenomena will be negligibly small, it can be assumed, in this case, that the effective diffusion coefficient through a partition with a single layer *D_ef_*_1_ will be the following:

(6) $$D_{ef1}=D\cdot \varepsilon$$

By calculating the effective diffusion coefficient on the basis of the relationship (4) and (5), and knowing the porosity of a single layer (0.008), it is possible to calculate the value of the diffusion coefficient *D*. Or, conversely, it is possible to count *D_ef_* by knowing the porosity of the medium and calculating the value of the coefficient *D* for free diffusion based on Stokes’ laws:

(7) $$D=\frac{k_{B}T}{6\pi \mu d}$$

where *k_B_*—Boltzmann constant, *T*—temperature, *μ*—fluid viscosity, and *d*—particle diameter.

Then, in order to calculate how the real porosity *ε_r_* changes upon diffusion through partitions with multiple layers, it is possible to use the calculated value of *D* on the basis of Equations (6) or (7) and use the following relationship:

(8) $$\varepsilon_{r}=\frac{D_{ef}}{D}$$

**Figure 9:**
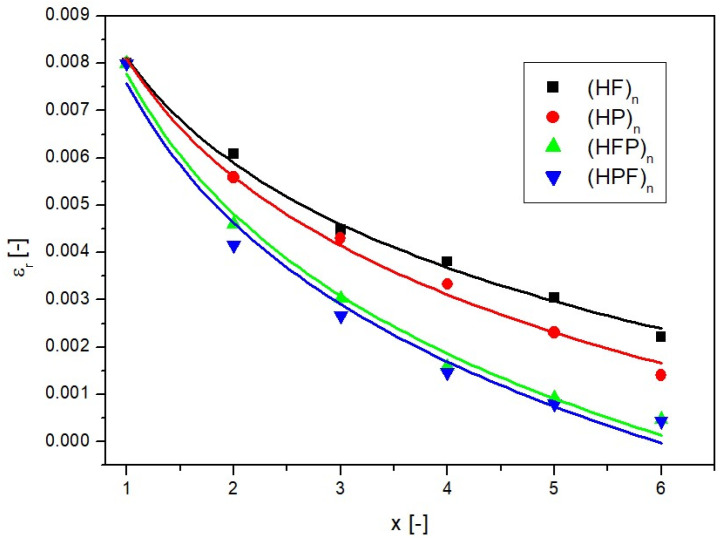
Dependence of real porosity on the number of layers for different filter combinations.

Following the calculation methodology outlined above, it was possible to determine the changes in real porosity relative to the number of layers, as shown in Figure 9.

As can be observed in Figure 9, the use of as few as six layers of alternating filters (with changing wettability) caused the active porosity to drop to almost zero. This means that the space through which nanoparticle transport took place was reduced almost completely. The change in actual porosity relative to the number of layers was described (continuous lines in Figure 9) by the following relationship:

(9) $$\varepsilon_{r}=x^{-k}+\varepsilon -1$$

where *k* is the parameter of the equation presented with the correlation coefficient in Table 1.

Combining relationship (8) and relationship (9), the relationship between the effective diffusion coefficient *D_ef_* and the number of layers can be found, which can be written with the following equation:

(10) $$D_{ef}=D\left(x^{-k}+\varepsilon -1\right)$$

The equation above can be checked for a single layer (*x* = 1), while *ε* can be calculated using Equation (6). Then, the effective diffusion coefficient can meet the equation $D_{ef}=D_{ef1}$.

Thus, it becomes most relevant to determine the parameter *k*. As read from the fit to the experimental data, it is larger for partitions with mixed wettability characteristics than for partitions with uniform wettability properties (see Table 1). Based on Equation (10), it can be deduced that when the parameter *k* is equal to 0, the effectiveness factor *D_ef_*, based on relation (6), will then take the value of the effectivity factor for a single layer *D_ef_*_1_. This is consistent with the assumption that with a single layer, any surface phenomena will be negligibly small.

**Figure 10:**
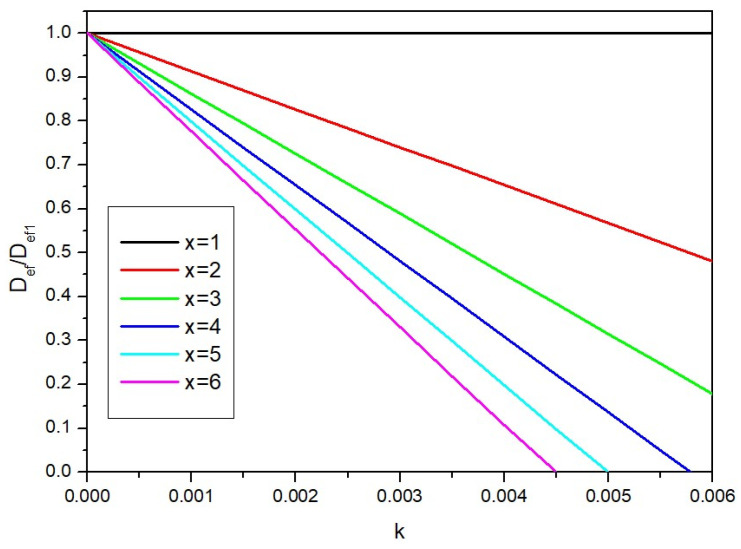
Dependence of the ratio of diffusion coefficients on the parameter *k.*

In order to trace how the value of the effective diffusion coefficient changes from the parameter *k* with different numbers of layers, a graph of the dependence of the ratio of diffusion coefficients *D_ef_*/*D_ef_*_1_ versus the parameter *k* was prepared, presented in Figure 10.

As can be observed in Figure 10, the effective diffusion coefficient decreases as the parameter *k* increases, and it is a linear decrease. The more layers the partition contains, the stronger the decrease. However, from a practical perspective, the most relevant question is how to determine the parameter *k*. For this purpose, we propose focusing on the extreme values.

According to the graph, there is a certain critical value of the parameter *k*, at which the ratio of the effective diffusion coefficient reaches zero; that is, there is no longer any diffusion, and the partition is an effective barrier to the transport of nanoparticles. Then, it can be written that *D_ef_* = 0; that is, according to Equation (10), the following notation is correct:

(11) $$0=D\left(x^{-k}+\varepsilon -1\right)$$

Based on experimental studies, it is possible to determine the number of layers *x* at which the diffusion process no longer occurs. For this purpose, diffusion tests can be carried out by adding more layers and checking for when the diffusion process can be observed. One can also run simulations and determine when the diffusion process is completely inhibited. It is also possible, based on knowledge of the structure under study, to assume a certain number of layers above which the diffusion process occurs negligibly slowly. When we find the number of layers at which the diffusion process, under given conditions, ceases to take place, we can insert the number of these layers as *x* into Equation (11). Knowing the porosity of a single layer, and the coefficient *D*, it is possible to determine the parameter *k* from this equation:

(12) $$k=-\frac{\log\left(1-\varepsilon \right)}{logx}$$

Having the parameter *k* determined, it is possible to determine the effective diffusion coefficient at any number of layers from Equation (10). In this way, how an effective diffusion coefficient depends on the porosity of the bed, the number of layers, and the surface phenomena occurring in them can be determined.

To test the reasoning above, the experimental data presented here were used. As can be observed for the (HPF)n partition, the use of the sixth layer almost completely eliminated the diffusion process; at the seventh layer, diffusion would not take place at all (see Figure 9). For the accuracy of the result, it was assumed that the critical number of layers at which diffusion does not occur is *x* = 6.5. By substituting this value into relation (12), a value of −0.00429 was obtained, which corresponds to the value in Table 1.

## 4. Materials and Methods

### 4.1. Characterization of Experimental Media

In order to evaluate the effect of variable wettability on the diffusion process of nanoparticles, a series of diffusion tests were carried out using a Franz chamber. Microfilters with modified wettability properties were used as filter barriers. SEFAR NITEX03-1/1 filters with hydrophilic properties and SEFAR NITEX03-1/1/h filters (Sefar, Poznań, Poland) exhibiting hydrophobic properties were used. The parameters of the filters used and the splicing method employed are shown in Table 2.

Nano-copper (nanoCu) particles were chosen for this study because they are becoming an active area of research in recent years due to their unique physical, chemical, electrical, and optical properties, in addition to their good antiseptic properties [24,25,26]. The main advantage of nanoCu is its low cost and availability compared to gold and silver nanoparticles, which allows for sample synthesis and a variety of nanoCu applications [27]. Among the most important features of nanoCu is its antibacterial, antiviral, and antifungal activities [28]. There are also numerous research papers outlining the use of nanoCu during cancer treatment [29,30,31]. In addition, studies have shown that nanoCu can be an effective alternative to conventional antibiotics, especially for treatment-resistant pathogens [32]. Due to their properties, nano-copper and its derivatives have gained applications in medicine not only for surface decontamination and wound care but also as drug carriers [24,27]. For example, nanoCu-based carriers have been used to treat skin infections caused by Staphylococcus aureus bacteria. An in vitro nanoCu release study showed a drug release rate of 96.5% [33]. Studies on the analysis of the transport of copper nanoparticles in skin structures can therefore contribute to the determination of optimal therapeutic conditions.

The study used aqueous solutions of nano-copper (nanoCu) particles as the test media. The nanoCu colloid used was a suspension of nanoparticles stabilized with water-soluble polyol polymers. The concentration of the base solution was 4000 ppm, but for testing, the solution was diluted 10-fold (400 ppm). The particles had a spherical shape, and dimensions were in the range of 5–12 nm, with an average particle diameter of 9 nm, according to the manufacturer’s characteristics (Limpio Company Grzybowo, Poland). The nanoCu used exhibited hydrophilic properties and Zeta potential <−25 (mV). The chemical composition of the nanoparticles was pure copper (Cu). The diluted solution (400 ppm) exhibited an electrical conductivity of 370 μS. UV-VIS studies showed a value of 562 nm (according to the manufacturer).

### 4.2. Experimental Procedure

**Figure 11:**
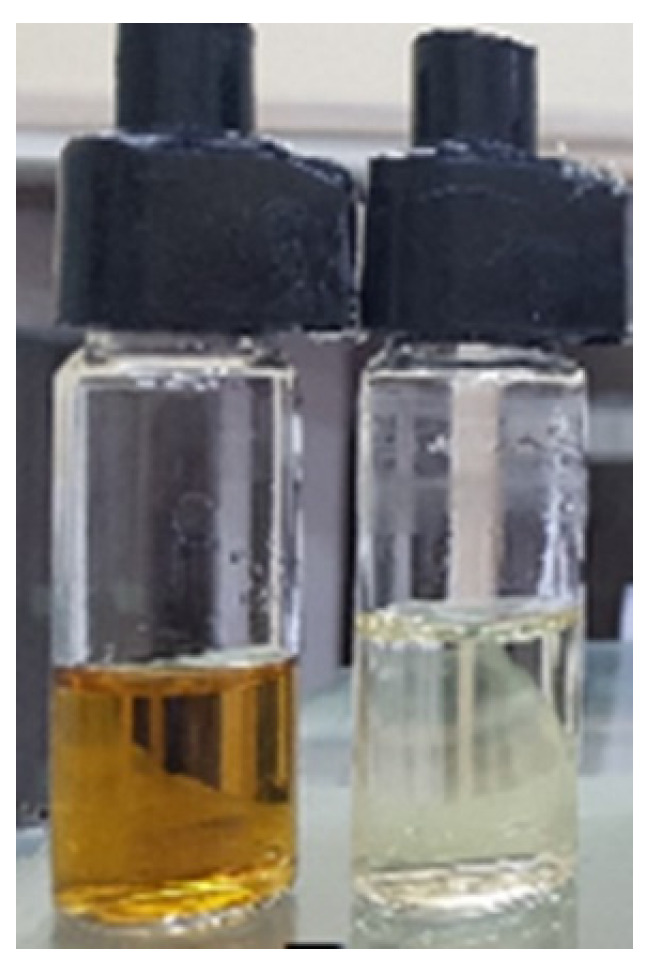
NanoCu solutions after the diffusion process: donor fluid (**left**); acceptor fluid (**right**).

The diffusion process was conducted using Franz diffusion chambers. The process was carried out for four different time periods. At the end of the duration, acceptor and donor fluids were analyzed for concentration. Examples of acceptor and donor fluid samples are shown in Figure 11. A conductometric method was used to analyze the concentration of copper nanoparticles in solution. Forced diffusion was conducted to significantly reduce the process time. For this purpose, a magnetic stirrer (Guardian 3000) (OHAUS Europe GmbH, Nänikon, Switzerland) was used at a speed of 150 rpm. The measurement times were chosen so that for the most optimal conditions (which was diffusion through a single hydrophilic filter), it was possible to reach a state close to equilibrium, which, in the assumed case, required 1200 s. In order to be able to study process changes over time, measurements were also carried out after 300 s, 600 s, and 900 s. All the conducted tests were repeated three times.

Studies of nanoparticle diffusion over time were carried out for both single layers of partitions and several layers—one on top of the other. Alternating systems of hydrophilic and hydrophobic partitions in various combinations were also used. This allowed us to determine changes in the concentration of nanoparticles in solution over time for the systems studied.

Initially, tests were performed for the diffusion of nanoparticles through single, double, and triple hydrophobic filters, then analogously through hydrophilic filters; then, tests were performed for alternate filters, with different combinations. The method of combining the filters is presented in the supporting figures next to the individual diagrams, and the colors indicate 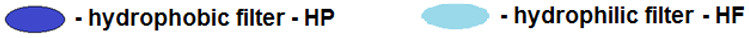.

It should be noted that the chosen media and the adopted research procedure provided a model research concept for capturing the effect of wettability and the number of layers on the diffusion of nanoparticles. Nevertheless, the results of these studies cannot directly relate to the study of diffusion in human skin, where the phenomena taking place are more complex and complicated.

## 5. Conclusions

Surface wettability plays an important role during the transport of various types of nanosystems through a variety of porous structures. The human skin is a structure that constitutes a great barrier against penetrating substances. The possibility of overcoming this offers excellent prospects for the development of methods for the non-invasive introduction of drugs into the human body. This paper presents an experimental study on the diffusion of nanoparticles through partitions with different wettability characteristics. We investigated how transport is affected by various alternating arrangements of hydrophobic and hydrophilic layers.

Diffusive transport through hydrophobic filters was less intense than through adequate hydrophilic filters. However, the greatest resistance to transport was the alternating configuration of hydrophobic and hydrophilic filters. In such a situation, with triple layers, more than a 40% lower concentration of nanoparticles in the acceptor solution was observed after diffusion than for the adequate process with hydrophilic filters. This means that alternate wettability properties play a large role in the transport of nanosystems through the given structures, and this must be taken into account when analyzing the movement of drug nanocarriers through variously lipophilic dermal layers.

Based on the results obtained, an attempt was made to present the mechanisms occurring during the movement of nanoparticles through structures with different wettability characteristics. A mathematical description of the process was also developed. On the basis of the proposed model approach and knowledge of the studied structure, it becomes possible to estimate the effect of layers’ alternating wettability characteristics on the transport of nanoparticles. With the assumptions obtained via the adopted model, it is possible to estimate the effective diffusion coefficient, taking into account the porosity of the structure and the influence of its wettability.

The results of this work offer the possibility of predicting how alternating hydrophobic and hydrophilic layers in the skin can affect the movement of a substance placed in a drug nanocarrier. Taking into account the influence of surface wettability in transdermal transport processes will allow for more precise drug delivery, which could significantly increase drug efficiency.

## Figures and Tables

**Table 1:** Parameters of Equations (2), (3), and (9).

	*a*	*n*	R^2^
HP	0.0188	0.3249	0.981
HF	0.0078	0.4842	0.975
HP+HF	0.0052	0.4866	0.978
HP3	0.0161	0.2673	0.974
HF3	0.0044	0.4618	0.977
HF-HP-HF	0.0027	0.4841	0.969
HP-HF-HP	0.0004	0.7533	0.956
HP-HF-HP-HF	0.0012	0.4499	0.994
HP-HF-HP-HF-HP	0.0001	0.7170	0.963
HP-HF-HP-HF-HP-HF	0.0002	0.8841	0.984
	***b***	***c***	**R^2^**
(HP)_n_	0.4959	0.0346	0.993
(HF)_n_	0.4506	0.0495	0.997
(HFP)_n_	0.4743	0.0793	0.984
(HPF)_n_	0.5394	0.0914	0.998
	***k***		**R^2^**
(HF)_n_	0.0032		0.994
(HP)_n_	0.0036		0.995
(HFP)_n_	0.0426		0.993
(HPF)_n_	0.0428		0.981

**Table 2:** Parameters of filters.

	Catalog No.	Mesh Size [μm]	Thread Diameter [μm]	Splice Density [n/cm]	Empty Area [%]	Splicing Method
Hydrophilic	SEFAR NITEX03-1/1	1	75	186	0.8	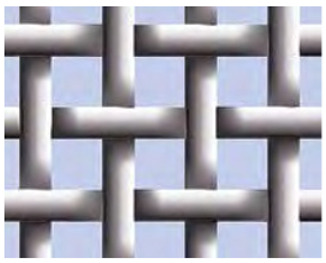
Hydrophobic	SEFAR NITEX03-1/1/h	1	75	186	0.8	

## Data Availability

Datasets are available on request from the authors.
